# Can Iron Absorption in Molasses Be Increased with Probiotic Additives? “Molasses with Increased Bioavailability”

**DOI:** 10.3390/nu17071150

**Published:** 2025-03-26

**Authors:** Yasin Yıldız, Atilla Topçu, Tolga Mercantepe, Medeni Arpa, İlknur Esen Yıldız, Levent Tümkaya

**Affiliations:** 1 Department of Paediatrics, Faculty of Medicine, Recep Tayyip Erdoğan University, 53200 Rize, Türkiye; 2 Department of Pharmacology, Faculty of Medicine, Recep Tayyip Erdoğan University, 53200 Rize, Türkiye; atilla.topcu@erdogan.edu.tr; 3 Department of Histology and Embryology, Faculty of Medicine, Recep Tayyip Erdoğan University, 53200 Rize, Türkiye; tolga.mercantepe@erdogan.edu.tr; 4 Department of Medical Biochemistry, Faculty of Medicine, Recep Tayyip Erdoğan University, 53200 Rize, Türkiye; medeni.arpa@erdogan.edu.tr; 5 Department of Infectious Diseases and Clinical Microbiology, Faculty of Medicine, Recep Tayyip Erdoğan University, 53200 Rize, Türkiye; ilknur.yildiz@erdogan.edu.tr; 6 Department of Histology and Embryology, Faculty of Medicine, Ondokuz Mayıs University, 55139 Rize, Türkiye; levent.tumkaya@omu.edu.tr

**Keywords:** molasses, probiotic, prebiotic, iron absorption

## Abstract

**Introduction:** There are many studies on the chemical and enzymatic interactions of probiotics, and the effects of *Lactobacillus plantarum* 299v on iron absorption have been clearly shown. The aim of this study was to investigate the effect of probiotics on the absorption of iron in molasses. **Material and method:** Wistar rats (*n* = 46) were taken four weeks after birth and divided into seven groups. Iron deficiency anemia was induced by giving “iron purified pellet” to the groups except the control group for four weeks and then the groups were given nutrients for eight weeks. In addition to iron deficiency anemia tests, immunohistochemical markers such as SCL11a, IRE1, Wnt2, and CD71 were examined. **Results:** The mean weight of the subjects was 309.5 ± 63.9 (226–424) g and no significant difference was observed in the laboratory values of metabolic data. When the laboratory values of iron deficiency anemia were examined, a statistically significant difference was found between the mean ferritin (*p* = 0.03) and hepcidin (*p* = 0.02) values of the groups. **Discussion:** Iron absorption analysis values were generally higher in the group receiving Fe^3+^ as expected. However, when the groups receiving molasses and additives were compared, the highest plasma iron level and Hb value were found in the *Lactobacillus plantarum* 299v group, and the highest ferritin and hepcidin levels were found in the Multiprobiotic group. No difference was observed between the body weights and fasting serum glucose levels of the groups despite daily molasses consumption, indicating the metabolic proactive effects of probiotics. **Conclusions:** Although no significant difference was detected between the groups receiving probiotics, iron absorption in molasses was increased with probiotic supplementation.

## 1. Introduction

Molasses is a syrup-like food obtained by converting rapidly perishable fresh fruits into a more durable product using various methods to extend their shelf life [1]. It has been produced for centuries all over the world by evaporating the juices of sugar-rich fruits such as grapes and mulberries [2]. Since molasses contains high amounts of sugar, mineral substances, and organic acids, it has a very important place in human nutrition and is a good source of energy. The amount of sugar in its content varies between 64.04 and 71.40% [3]. In addition, molasses contains varying amounts of proteins, amino acids, flavonoids, and phenolic compounds in its composition depending on the fruit used in production [1]. Molasses also contains vitamins such as B1 (thiamine), B2 (riboflavin), B5 (pantothenic acid), folic acid, vitamin C (ascorbic acid), and minerals such as Fe, Cu, Zn, K, Ca, Na, Mg, and P [4].

Iron deficiency anemia (IDA) is an important public health problem affecting an estimated 136 million children and 264 million women worldwide. The most common cause of iron deficiency anemia in the community is intake deficiency (malnutrition, iron-poor diet, modern diet, and factors that reduce absorption) and the aim of treatment is to increase the amount of iron intake. Chemical iron preparations (Fe^2+^ and Fe^3+^) are generally used in the treatment of IDA. However, this treatment method has side effects on many systems and organs, particularly the gastrointestinal system, and its tolerance is very difficult, especially in the pediatric population [5]. In order to reduce side effects or increase absorption; different formulations (microsomal iron, liposomal iron, etc.) and trials with different additives (probiotics, vitamins, etc.) are ongoing [6].

The positive effects of probiotics on the absorption of chemical iron preparations have been investigated by many scientific studies in the past. Numerous studies have been conducted with single or multiple probiotics with microorganisms such as *Escherichia coli Nissle* 1917, *Lactobacillus plantarum* 299v, *Lactobacillus casei*, *Lactobacillus acidophilus* [7,8]. These studies have been conducted in both experimental animals and humans, in different age and gender groups, with chemical iron preparations and different foods. A meta-analysis was published on a total of 12 human studies with different probiotics and it was shown that *Lactobacillus plantarum* 299v provided a significant increase in iron absorption [9].

Although studies with foodstuffs such as milk, wheat porridge, fruit drinks, and breakfast cereals, as well as chemical iron preparations, were found in the literature to increase iron intake, no studies were found involving molasses despite its high iron content. Therefore, the aim of our study was to observe whether supplementation of grape molasses with probiotics would alter iron absorption.

## 2. Method

**Experimental Animals**: A total of 46 male and female Sprague Dawley rats aged 16 weeks and weighing 300–350 g were used in the study. All the animals were treated in accordance with the *Care and Use of Laboratory Animals* protocols in both National and International Research Council Guidelines. All of the animals were kept in standard cages consisting of transparent polycarbonate material with sawdust on the floor, in an environment of 20–23 °C and 55–65% humidity, especially in standard lighting conditions (12 h dark: 12 h light) before and during the study. The rats were allowed ad libitum access to standard rat feed, tap water, and only drinking water during the study period. Ethical approval for the study was obtained from the Recep Tayyip Erdogan University, Animal Experiments Local Ethics Committee (Approval Number: 2020/49-31 December 2020).

**Scheme 1:**
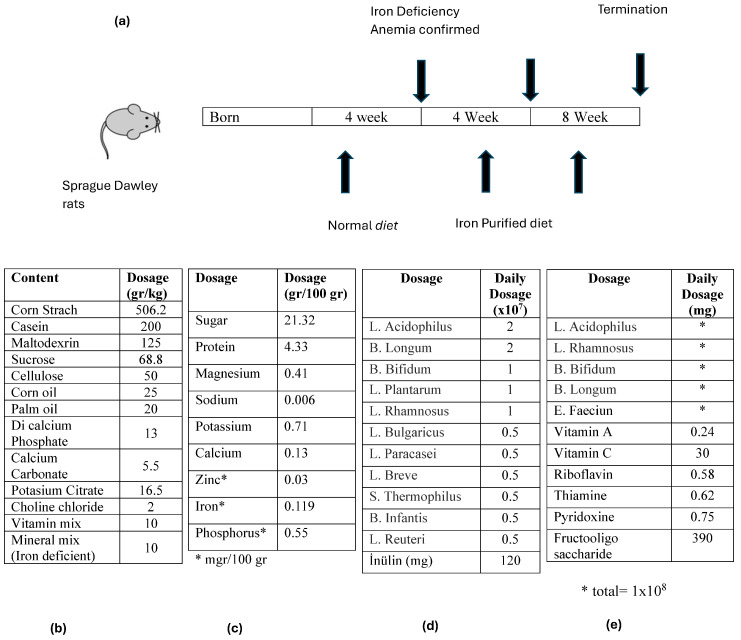
The timeline of the study and the characteristics of the food supplements used: (**a**) the experimental animals were given iron purified diet for 4 weeks after birth and then iron purified diet and food supplements for 8 weeks after the occurrence of iron deficiency anemia was confirmed, (**b**) chemical content of iron purified diet, (**c**) chemical content of molasses, (**d**) content of polyprobiotic supplement given to Group 6, and (**e**) content of the polyprobiotic supplement given to Group 7.

**Identification of groups:** Sprague Dawley rats were included in the study and divided into seven groups (Table 1). At baseline, 4-week-old rats were kept in separate cages and fed an iron-poor diet except for the control group. The study schedule is given in Scheme 1a. For this purpose, the rats in the experimental group were given 20 g/day of “iron-deficient purified feed” pellets (the pellet content is given in Scheme 1b). The control group received 20 g/day of conventional pellets. After four weeks of iron-poor diet, the blood values of the rats were measured to check whether iron deficiency anemia occurred. After it was determined that the rats developed IDA, rats except the control group were given an iron-poor diet and nutrients shown in Table 1 for eight weeks [8,10,11,12,13].

## 3. Procedures

**Preparation procedure of iron-deficient purified pellet, Iron, Molasses, and Probiotics:** The rats in the experimental group were fed with specially prepared “iron-deficient purified feed” (Arden Research and Experiment LTD, Ankara, Turkey). The feed content is given in Scheme 1b. Ferrum Hausmann 50 mg/mL Drop (Abdi İbrahim İlaç Sanayii/Turkey) containing Demir III Hydroxide Polymaltose was used as iron preparation. A total of 1 cc solution containing 2 mg/kg iron (+3 valence) was given by gavage once a day per rat. Black grape extract from Malatya/Turkey was used as molasses and its chemical content is given in Scheme 1c. It was given 1 cc per rat once a day by gavage. As monoprobiotic supplement (Group 5); 1 × 10^8^ *Lactobacillus plantarum* 299v. (Innovall Microbiotic RDS capsules, WEBER & WEBER GmbH & Co. KG) was made into an aqueous solution and given by gavage once a day. As a polyprobiotic supplement (Group 6), the rats were given 1 × 10^8^ probiotic mixture (Probest Dıgestıve Probiotik, Abdi İbrahim İlaç Sanayii, Turkey) once a day by gavage. Its content is given in Scheme 1d. The last group (Group 7) was given a polyprobiotic (1 × 10^8^) + vitamin combination (NBL Probiotic Gold, Nobel İlaç Sanayii, Turkey). Its content is given in Scheme 1e.

**Procedure for biochemical analysis of serum samples:** Blood samples were collected in tubes containing EDTA and analyzed for blood count (Mindray BC6000 Hematology Analyzer, Shenzhen, China). The samples collected in biochemistry tubes after centrifugation were then analyzed for iron, iron-binding capacity, cholesterol, triglycerides, HDL, cholesterol, triglycerides, and iron by the spectrophotometric method and Chemiluminescence Microparticle Immunoassay method. After the collection of the samples was completed, these plasma samples were manually analyzed for hepcidin, Wnt2b, and Anti-IRE1 by the Elisa method.

**Histochemical analysis procedure:** Intestinal tissue materials removed from the rats were trimmed to a volume of 1.5 cm^3^. The trimmed intestinal tissue samples were fixed with Bouin’s solution for 24 h for histopathological analysis (Merck GmbH, Darmstadt, Germany). After fixation, routine histologic tissue follow-up was performed on an automatic tissue tracking device (Shandon, Citedal 2000, Helensburgh, UK). In this context, the intestinal tissue samples were dehydrated with increasing alcohol series (50%, 60%, 70%, 70%, 80%, 90%, and 100%; Merck GmbH, Darmstadt, Germany). The intestinal tissue samples were then soaked in two series of xylol solution (Merck, Darmstadt, Germany) and mordanted. In the next step, the intestinal tissue samples were kept in soft paraffin (42–44 °C, Merck GmbH, Darmstadt, Germany) for 1 h and hard paraffin (54–56 °C, Merck GmbH, Darmstadt, Germany) overnight and inclusion was performed. The samples of intestinal tissues were then paraffin-blocked with a tissue embedding dispenser (Leica EG1150 C, Leica Biosystems, Nußloch, Germany). The paraffin blocks of the intestinal tissues were sectioned at 4–5 μm thickness with a rotary microtome (Leica, RM2125RT, Leica Biosystems, Germany) and stained with hematoxylin (Harris hematoxylin, Merck GmbH, Germany) and Eosin (H&E) (Eosin G, Merck, Germany). The preparations were examined under a light microscope (Olympus BX51, Olympus Corporation, Tokyo, Japan) and photographed with an Olympus DP71 camera (Olympus Corporation, Tokyo, Japan).

**Immunohistochemical (IHC) Analysis Procedure:** The samples of intestinal tissue removed from the rats for immunohistochemical analysis were fixed with 10% neutral formalin stabilized with methyl alcohol for 24 h (Sigma Aldrich, St. Louis, MO, USA). After fixation, paraffin blocks of intestinal tissue were subjected to routine histologic follow-up procedures, and 3–5 μm thick sections were taken on positively charged slides (PatoLab, Istanbul, Turkey). The sections were immunohistochemically stained with primary antibodies using an automatic immunohistochemistry staining device (Leica BOND-MAX IHC/ISH, Leica Biosystems, Australia) in accordance with the manufacturer’s procedures. Mucosal damage and immune positivity were scored (Table 2a,b).

**Statistical Analysis**: Data obtained from the quantitative and semi-quantitative analyses were tested for normality by Shapiro–Wilk’s test, Q-Q plot, Skewness–Kurtosis values, and Levene’s tests using the SPSS 18.0 statistical software (IBM Corp., Armonk, NY, USA). The conformity of the parameters of the study groups to normal distribution was evaluated by Kolmogorow–Simirnow test. Intergroup parameters were compared according to the Student t-test or Mann–Whitney test, and the relationships between intragroup parameters were analyzed by Pearson or Spearman correlation test. Parametric data obtained from the analyses were calculated as mean ± standard deviation and differences between the groups were compared by one-way analysis of variance (ANOVA) followed by Duncan’s test. *p* < 0.05 was considered significant. Non-parametric data from the semi-quantitative analysis were calculated as a median with an interquartile range of 25% and 75%. Nonparametric data were analyzed using the Mann–Whitney U test and Bonferroni correction test. Parametric data from quantitative analysis were calculated as standard deviation and differences between groups were subjected to one-way analysis of variance followed by Bonferroni correction test.

## 4. Results

The mean weight of 46 rats was 309.5 ± 63.9 (226–424) g at the end of the experiment. Among the experimental groups, the highest mean weight was observed in Group 7 with 325.7 ± 43.2 (281–380) g, and the lowest mean weight was observed in Group 5 with 286.6 ± 69.5 (228–399) g and no statistically significant difference was observed (*p* = 0.83). When the metabolic tests of the subjects were analyzed, the mean glucose levels were 164.6 ± 28.1 (97–295) mg/dL, mean triglyceride levels were 47.7 ± 19.4 (25–118) mg/dL, mean HDL cholesterol levels were 48.2 ± 11.9 (33–89) mg/dL, and no statistically significant difference was observed except for the triglyceride level (*p* = 0.02). The values of the metabolic tests of all the groups are given in Table 3.

When the laboratory values of iron deficiency anemia of the subjects were examined, the mean iron levels of the groups were 229.1 ± 76.0 (112–355) mg/dL. When the control group was excluded, the highest mean iron level was observed in Group 3 with 276.7 ± 84.0 (168–355) mg/dL, and the lowest was observed in Group 2 with 182.0 ± 66.8 mg/dL (112–265) (*p* = 0.41). The mean hemoglobin levels of the groups were 13.9 ± 1.1 g/dL (10.1–16.6). No statistically significant difference was found between the laboratory values of iron deficiency anemia (Table 4).

**Figure 1:**
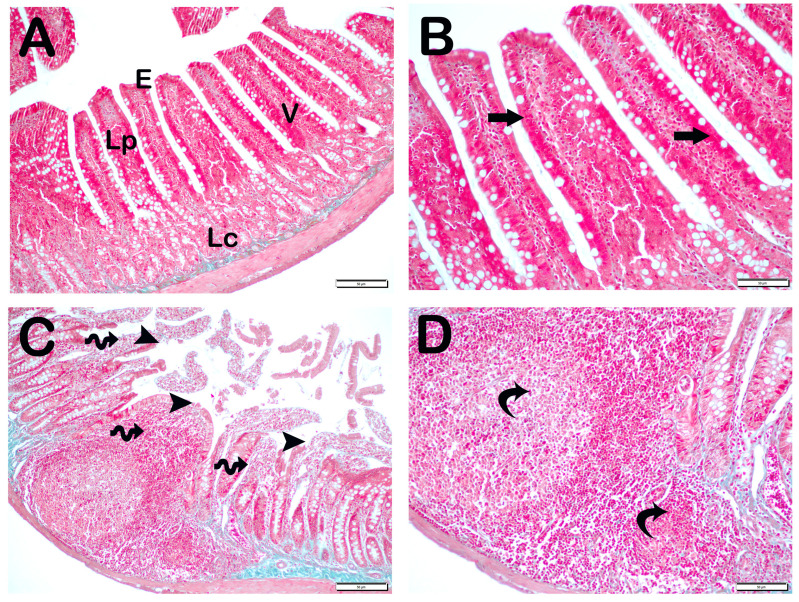

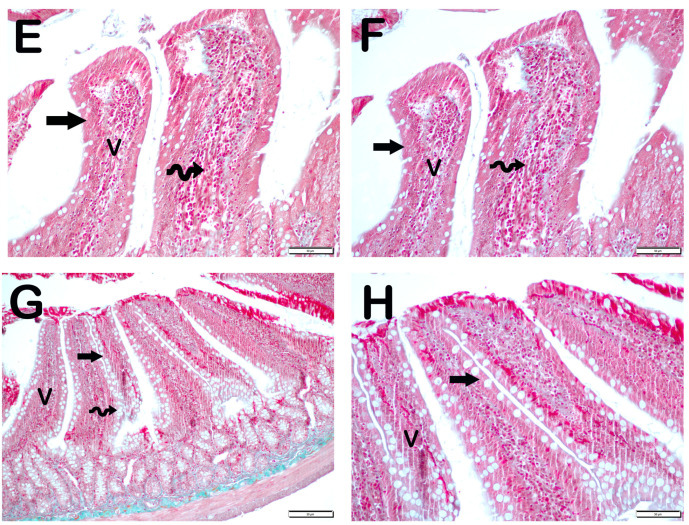

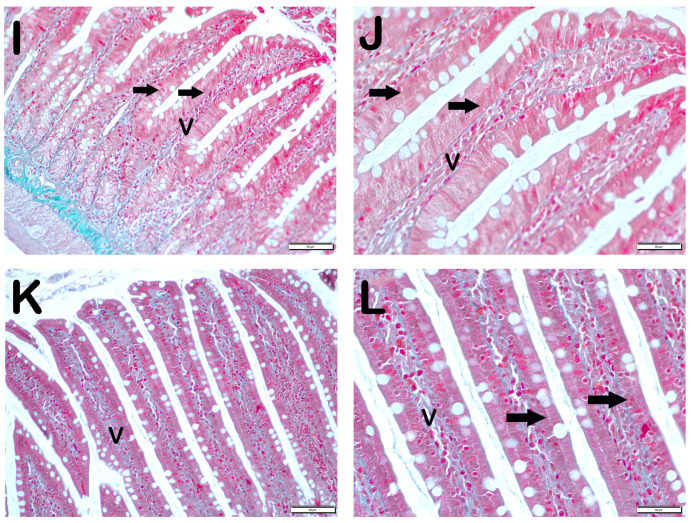
Representative light microscopic pictures of small intestinal sections stained with Goldners Masson Trichrome stain. Villus (V), Lieberkühn crypt (Lc), Lamia propria (Lp), epithelium (E). (**A**) (×200), (**B**) (×400) Group 1: In the intestinal tissue sections of the control group, a mucosa (arrow) structure consisting of single-layer prismatic epithelium with typical villus structures is observed (MHDS: 0 (0–0)). (**C**) (×200), (**D**) (×400) Group 2: Sloughing of single-layer prismatic cells with microvilli (arrowhead) and villar fusions (spiral arrow) are observed in the villi. In addition, diffuse inflammations (curved arrows) are observed in the lamina propria (MHDS: 3.5 (3–4)). (**E**) (×20), (**F**) (×40) Group 3: Epithelial shedding (arrow), villus loss (spiral arrow) and reduction in inflammation are observed. (MHDS: 2 (2–2)). (**G**) (×200), (**H**) (×400) Group 4: Sloughing of enterocytes (arrow) and decrease in interstitial inflammation (spiral arrow) are observed (MHDS: 2(1–2)). (**I**) (×200), (**J**) (×400) Group 5: Single-layer prismatic epithelial cells (arrow) with typical villus structure are observed (MHDS: 2(2–1)). (**K**) (×200), (**L**) (×400) Group 6: Small intestinal tissue sections show villi with typical enterocytes and lamina propria (Lp) structures (MHDS: 1 (1–2)). (**K**) (×200), (**L**) (×400) Group 7: Reduced epithelial shedding (arrow), villar fusion, and interstitial inflammation, accompanied by diffuse villi composed of typical enterocytes and lamina propria (Lp) structures (MHDS: 1(1–1)).

**Histopathological Analysis**: The mucosal histopathological damage scores (MHDSs) for the groups are given in Table 5. Light microscopy revealed that the villi in the control group (Group 1) were covered by a single layer of prismatic epithelial cells with a normal structure, and the Lieberkühn crypts were prominent (Figure 1A,B, MHDS: 0 (0–0)). In contrast, in the small intestinal tissue sections of the anemia group (Group 2), the number of villi was decreased and villi fusions were common. In addition, there were lesions in the duodenal mucosa accompanied by epithelial shedding and diffuse edematous areas in the lamina propria (Figure 1C,D, *p* = 0.001, MHDS: 3.5 (3–4)). In contrast, Group 3’s small bowel tissue sections showed a decrease in epithelial loss, villar junctions, and infiltrative areas in the duodenal mucosa (Figure 1E,F, *p* = 0.001, MHDS: 2 (2–2)). Compared to Group 1, Group 2, Group 4, and Group 5 showed a decrease in villar fusion, enterocyte loss, and inflammation in the lamina propria (Figure 1E–H, *p* = 0.00, *p* = 0.001 MHDS: 2 (1–2), MHDS: 2 (1–2), respectively). Similarly, duodenal ulceration associated with enterocyte loss and decreased villar fusion were observed in Group 6 and Group 7 compared to Group 2. In addition, there was a decrease in infiltrative areas in the lamina propria. Furthermore, in Group 7, the villar fusion and interstitial inflammations were statistically lower compared to Group 4 and Group 5 treatment groups (Figure 1G–J, *p* = 0.001, *p* = 0.001, MHDS: 1 (1–2), MHDS: 1 (1–1), respectively) (Table 5).

## 5. Quantitative Analysis

**Figure 2:**
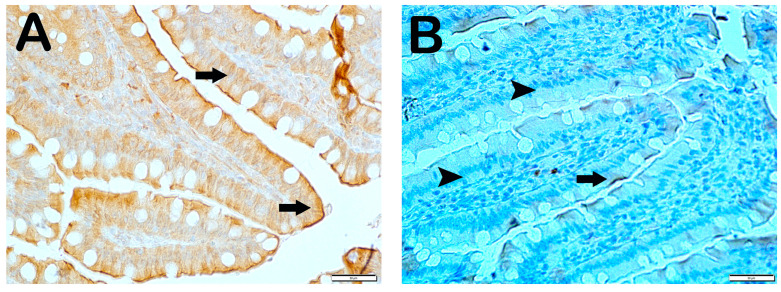

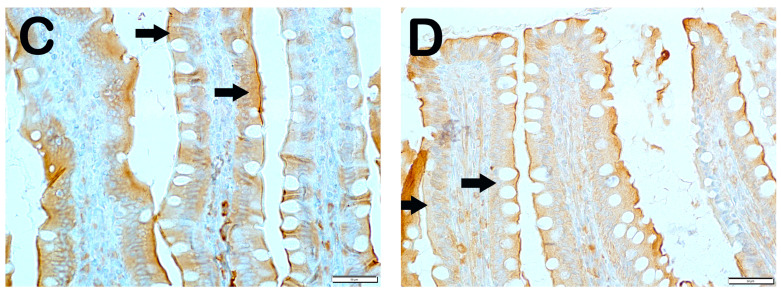

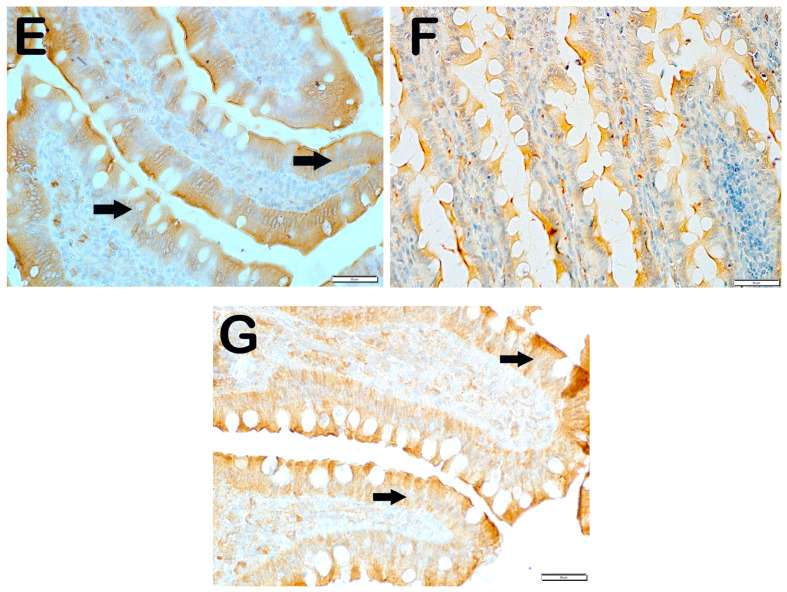
Representative light microscopic pictures of small intestinal tissue sections incubated with Scl11a primary antibody. (**A**) **(×400) Group 1:** Normal single-layer prismatic epithelial cells with microvilli showing intense Scl11a positivity (arrow). (**B**) **(×400) Group 2:** Single-layer prismatic epithelial cells (arrow) with microvilli show decreased Scl11a positivity (arrowhead). (**C**) **(×40) Group 3:** An increased number of cells showing Scl11a positivity is observed in epithelial cells in the villi (arrow). (**D**) **(×400) Group 4:** Epithelial cells in the villi show a large number of cells showing Scl11a positivity (arrow). (**E**) **(×400) Group 5:** Intense immunopositivity is observed in single-layer prismatic epithelial cells. (arrow) (**F**) **(×400) Group 6:** Enterocytes showing intense immunopositivity are observed to be increased in number (**G**) **(×400) Group 7:** Intense immunopositivity is observed in enterocytes in large numbers forming the epithelium of the villi (arrow).

**SCL11a positivity:** When samples of small intestinal tissue incubated with SCL11a primary antibody were examined under light microscopy, it was found that the number of enterocytes showing SCL11a positivity, which was 62.6 ± 4.02 per mm^3^ in Group 1, decreased compared to 16.8 ± 4.19 in Group 2 (Table 6, *p* = 0.001). In contrast, the numerical density of SCL11a positive enterocytes, which was 16.8 ± 4.19 per mm^3^ in Group 2, increased to 38.3 ± 3.46 in Group 3 (Table 6, *p* = 0.001). Similarly, the numerical density of SCL11a positive enterocytes measured in Group 2 was increased in Group 4 and Group 5 (16.8 ± 4.19, 41.65 ± 3.99, and 44.15 ± 5.73 per mm^3^, respectively) (Table 6, *p* = 0.001, *p* = 0.001, respectively). Similarly, the numerical density of SCL11a positive enterocytes measured in Group 2 was increased in Group 6 and Group 7, respectively (16.8 ± 4.19, 52.2 ± 4.2, and 55.85 ± 3.44 per mm^3^, respectively) (Table 6, *p* = 0.001, *p* = 0.001, respectively). In addition, the numerical density of SCL11a positive enterocytes measured in Group 4 and Group 5 was higher in Group 6 (41.65 ± 3.99, 44.15 ± 5.73, and 52.2 ± 4.2, respectively) (Table 6, *p* = 0.001, *p* = 0.001, respectively) (Figure 2).

**Figure 3:**
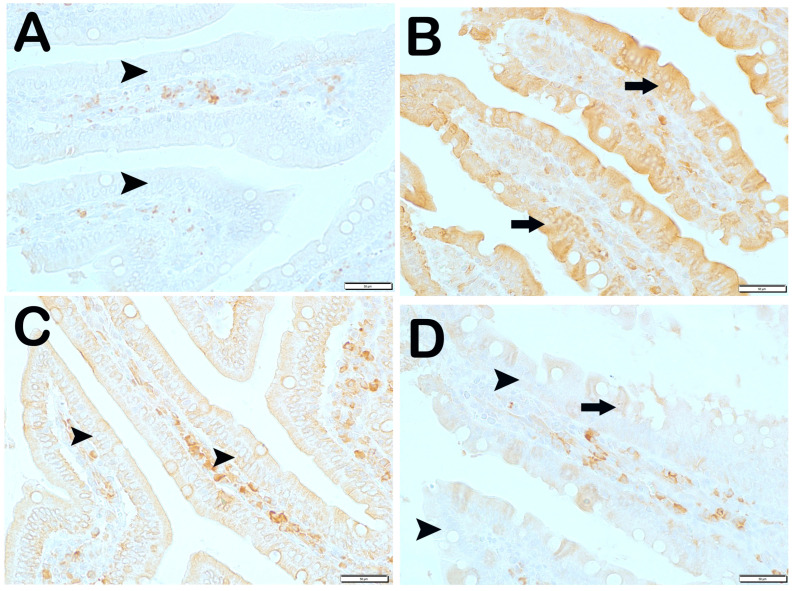

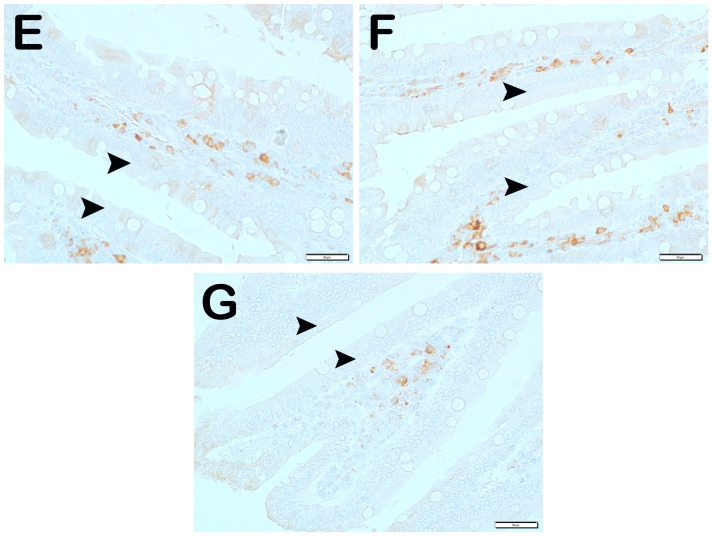
Representative light microscopic pictures of small intestinal tissue sections incubated with IRE1 primary antibody. (**A**) **(×400) Group 1:** Normal enterocytes and IRE1-negative enterocytes are observed in the small intestine sections of the control group (arrowhead). (**B**) **(×400) Group 2:** Enterocytes showing intense IRE1 positivity are observed to be common (arrow). (**C**) **(×400) Group 3:** Enterocytes showing IRE1 positivity were observed to be reduced in number (arrowhead). Immune-negative cells (arrowhead). (**D**) **(×400) Group 4:** Enterocytes showing IRE1 positivity in the villi are decreased (arrow). Immune-negative cells (arrowhead). (**E**) **(×400) Group 5:** Enterocytes in the villi are observed to be diffusely immune-negative (arrowhead). (**F**) **(×400) Group 6:** Enterocytes showing intense IRE1 positivity are decreased in number (arrowhead). (**G**) **(×400) Group 7:** Enterocytes showing intense IRE1 positivity were decreased in number (arrowhead).

**IRE1 positivity:** When the samples of small intestinal tissue incubated with IRE1 primary antibody were examined under the light microscope, we found that the number of enterocytes which was 11.7 ± 2.68 per mm^3^ in the control group showing IRE1 positivity increased to 66.5 ± 92.26 in the anemia group (Table 6, *p* = 0.001). On the contrary, we observed that the numerical density of IRE1 positive enterocytes measured as 66.5 ± 92.26 per mm3 in the anemia group decreased to 20.4 ± 3.81 in the anemia + Fe group (Table 6, *p* = 0.001). Similarly, we observed that the numerical density of enterocytes showing IRE1 positivity, which was measured as 66.5 ± 92.26 per mm3 in the anemia group, decreased to 21.30 ± 4.81 in the anemia + P group and 17.25 ± 1.83 in the anemia + P+P1 group, respectively (Table 4, *p* = 0.001, *p* = 0.001). Similarly, we found that the numerical density of IRE1 positive enterocytes measured as 66.5 ± 92.26 per mm^3^ in the anemia group decreased to 10.4 ± 2.98 in the anemia + P + M1 group and 9.15 ± 2.13 in the anemia + P + M2 group, respectively (Table 6, respectively; *p* = 0.001, *p* = 0.001) (Figure 3).

**Figure 4:**
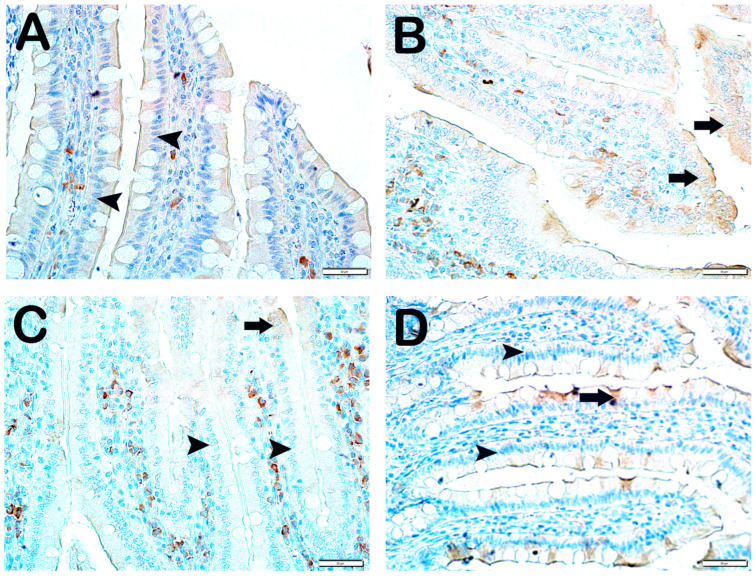

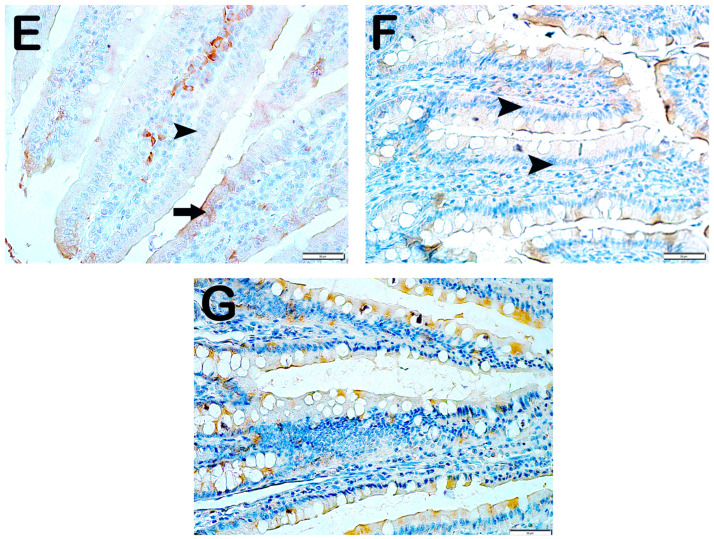
Representative light microscopic pictures of small intestinal tissue sections incubated with Wnt2 primary antibody. (**A**) **(×400) Group 1:** In the small intestine sections of the control group, Wnt2-negative enterocytes with normal structure are observed (arrowhead). (**B**) **(×400) Group 2:** Enterocytes showing intense Wnt2 positivity are observed to be common (arrow). (**C**) **(×400) Group 3:** Enterocytes showing Wnt2 positivity were observed to be reduced in number (arrow). Immune-negative cells (arrowhead). (**D**) **(×400) Group 4:** Enterocytes showing Wnt2 positivity in the villi are decreased (arrow). Immune-negative cells (arrowhead). (**E**) **(×400) Anemia Group 5:** Wnt2-negative enterocytes (arrow) in the villi are commonly observed (arrowhead). (**F**) **(×400) Group 6:** Enterocytes showing intense immunopositivity are reduced in number (arrowhead). (**G**) **(×400) Group 7:** Enterocytes showing intense Wnt2 positivity are reduced in number.

**Wnt2 Positivity:** When the samples of small intestinal tissue incubated with Wnt2 primary antibody were examined under the light microscope, it was observed that the number of immunopositive enterocytes showing Wnt2 positivity increased from 6.9 ± 2.75 per mm^3^ in Group 1 to 28.4 ± 6.26 in Group 2 (Table 6, *p* = 0.001). In contrast, we observed that the numerical density of Wnt2 positive enterocytes in Group 2 decreased in Group 3 (28.4 ± 6.26 and 12.7 ± 2.34 per mm^3^, respectively) (Table 6, *p* = 0.001). Similarly, we observed that the numerical density of enterocytes showing Wnt2 positivity in Group 2 was decreased in Group 4 and Group 5 (28.4 ± 6.26, 12.95 ± 1.57, and 13.45 ± 1.67 per mm^3^, respectively) (Table 6, *p* = 0.001, *p* = 0.001, respectively). Similarly, the numerical density of Wnt2 positive enterocytes in Group 2 was found to be decreased in Group 6 and Group 7, respectively (28.4 ± 6.26, 10.55 ± 1.87, and 9.3 ± 2.15 per mm^3^) (Table 6, *p* = 0.005, *p* = 0.001, respectively) (Figure 4).

**Figure 5:**
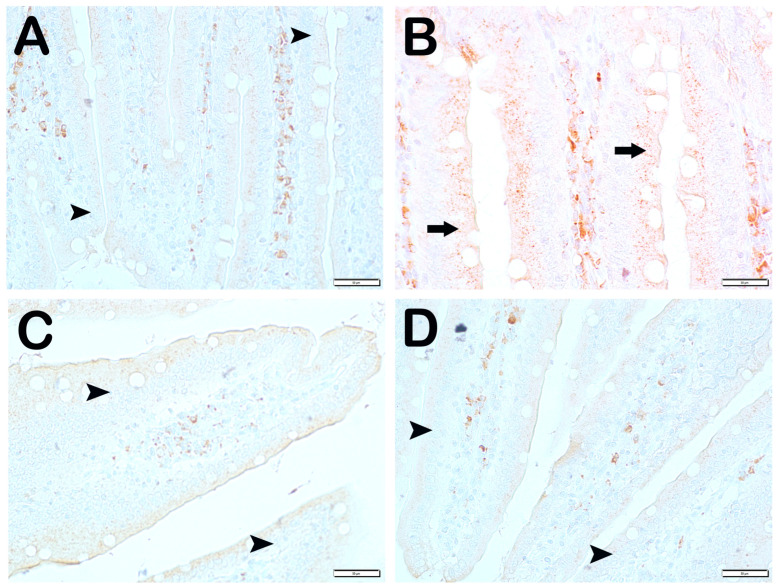

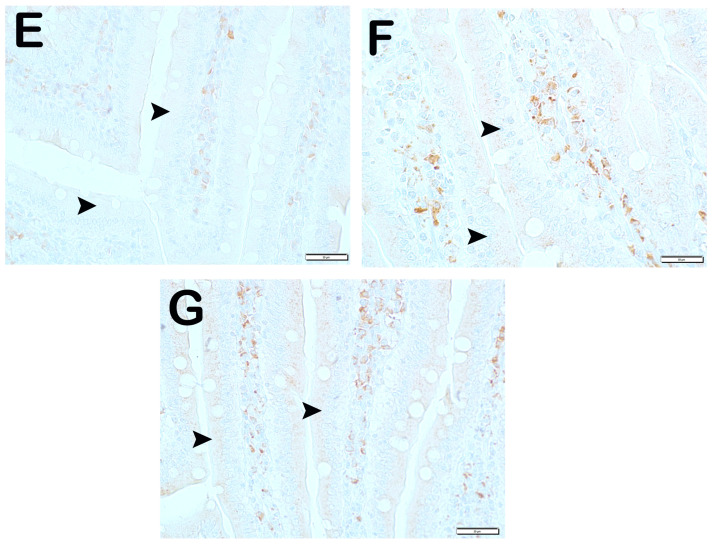
Representative light microscopic images of small intestinal tissue sections incubated with CD71 primary antibody. (**A**) **(×400) Group 1:** CD71-negative enterocytes are observed in normal villi of the control group (arrowhead). (**B**) **(×400) Group 2:** Enterocytes showing intense Wnt2 positivity are observed in the villi (arrow). (**C**) **(×400) Group 3:** Enterocytes showing intense immunopositivity are reduced in number (arrow). Immune-negative cells (arrowhead). (**D**) **(×400) Group 4:** The number of enterocytes showing immunopositivity in the villi is decreased. Immune-negative cells (arrowhead). (**E**) **(×400) Group 5:** CD71-negative enterocytes are commonly observed in thin nares tissue sections (arrowhead). (**F**) **(×400) Group 6:** Enterocytes showing intense CD71 positivity were reduced in number (arrowhead). (**G**) **(×400) Group 7:** Enterocytes showing CD71 positivity are reduced in number (arrowhead).

**CD71 Positivity:** When the samples of small intestinal tissue incubated with CD71 primary antibody were examined under the light microscope, it was observed that the number of immunopositive enterocytes showing CD71 positivity increased from 4.7 ± 0.65 per mm^3^ in Group 1 to 22.95 ± 8.89 in Group 2 (Table 6, *p* = 0.001). In contrast, the numerical density of CD71 positive enterocytes in Group 2 decreased in Group 3 (22.95 ± 8.89 and 14.6 ± 1.78 per mm^3^, respectively) (Table 6, *p* = 0.001). Similarly, the numerical density of enterocytes showing CD71 positivity in Group 2 was decreased in Group 4 and Group 5 (22.95 ± 8.89, 15.45 ± 1.79, and 9.5 ± 2.21 per mm^3^, respectively) (Table 6, *p* = 0.001, *p* = 0.001, respectively). Similarly, the numerical density of CD71 positive enterocytes in Group 2 was found to be decreased in Group 6 and Group 7 (22.95 ± 8.89, 10.35 ± 2.27, and 9.2 ± 2.91 per mm^3^, respectively) (Table 6, *p* = 0.005, *p* = 0.001, respectively). In addition, the numerical density of CD71 positive enterocytes was higher in Group 3 (14.6 ± 1.78) and Group 4 (15.45 ± 1.79) than in Group 6 (10.35 ± 2.27) (Table 6, *p* = 0.01, *p* = 0.001, respectively) (Figure 5).

## 6. Discussion

Studies examining the chemical and enzymatic interactions of probiotics with iron-containing drugs and foods have demonstrated an increase in iron absorption. In our study, which we conducted with the hypothesis that iron absorption could be enhanced by adding different probiotic additives to molasses, we found that iron absorption values were higher in the groups given probiotic additives compared to the group fed only molasses.

In general, glucose and fructose, the major carbohydrates of molasses, can account for more than 60% of the dry weight of molasses [14]. High doses of fructose intake can induce the development of metabolic syndrome and lead to non-alcoholic fatty liver disease [15,16,17]. This high glucose and fructose content may be considered as a limiting factor in molasses consumption. In our study, no statistically significant difference was observed in the average body weights of the rats, despite daily administration of molasses equivalent to 0.5% of their average body weight. Interestingly, the lowest mean body weight was observed in Group 5 (molasses + *Lactobacillus plantarum* 299v). In a meta-analysis, Lactobacilli have been shown to reduce body weight and/or body fat [18]. In our study, no significant difference was observed when the groups were compared in terms of ALT and AST levels. Similarly, when all the groups were compared in terms of LDL cholesterol, triglyceride, and total cholesterol values, no significant difference was observed. It is noteworthy that the mean total and LDL cholesterol values of the polyprobiotic group (Group 6) were the lowest among all the groups. These results show the positive effects of probiotics on lipid panels in the groups receiving molasses. In a meta-analysis investigating the effect of probiotics on lipid profiles in hypercholesterolemic adults, as in our study, no significant effect of probiotics on triglyceride and HDL-C levels was found, but it was shown that probiotics could significantly decrease the total cholesterol and LDL-C levels [19]. The biomarkers we used in our study do not fully reflect liver pathology, especially in early-stage or mild cases of NAFLD. Histological examination of liver tissue is considered the gold standard for the diagnosis of NAFLD, as it can assess the presence of hepatic steatosis, inflammation, and fibrosis, which are key to understanding the condition of the liver. Without histopathological analysis, it is difficult to fully interpret the safety and efficacy of the intervention, particularly in terms of its effects on liver health. The limitations of our study include the lack of histopathological analysis of the liver.

Side effects in the prophylaxis or treatment of IDA are dose-dependent. The local side effects of treatment have been reported in other systems, particularly the gastrointestinal tract (iron toxicity). An increase in Crohn’s disease and its severity has been found in rats receiving long-term iron therapy [20]. Unabsorbed dietary iron has been shown to cause disruption in the intestinal microbiota (dysbiosis) and alter the ratio of protective to pathogenic bacteria [21]. In neurodegenerative disorders (e.g., Alzheimer’s and Parkinson’s), osteoarthritis, and idiopathic pulmonary fibrosis, “iron overload” has been observed to be associated with disease severity [22]. For all these reasons, studies aim to “increase iron bioavailability” instead of “high-dose iron therapy”. In the study conducted with *Escherichia coli* Nissle 1917, it was observed that transferrin-bound iron levels were higher in the probiotic group compared to the control group. It was also shown that compared to the control group, serum and hepatic lipid, blood glucose, and antioxidant levels in blood and liver, as well as body weight improved [8]. It has been shown that iron metabolism was impaired in mice whose intestinal microbiota was sterilized with low-dose radiation, the intestinal cells of these mice were iron deficient, and epithelial cells supported iron storage following microbial colonization [13]. In our study, when the laboratory data of iron metabolism were analyzed, in general, all the parameters were found to be higher in the Fe^3+^-supplemented (Group 3) and control (Group 1) groups as expected. However, when the molasses and probiotic groups were compared, the highest plasma iron and Hb values were observed in Group 5. According to a meta-analysis examining the effects of *Lactobacillus plantarum* 299v on iron absorption, a statistically significant increase in iron absorption was found among those receiving Lp299v [9]. All eight studies using a random-effects model demonstrated a significant increase in iron absorption following administration of the probiotic Lp299v with a pooled SMD (an average intervention effect size) of 0.55 (*p* = 0.001) [9].

Solute Carrier Family 11, Member 2 (SLC11A2) functions as a proton-dependent iron importer of Fe^2+^ [23]. It has been suggested that iron deficiency can activate the Inositol-requiring enzyme 1 (IRE1) signaling pathway, thus contributing to pathological changes in tissues [24,25]. Iron overload has been reported to regulate WNT signaling, while iron chelation inhibits it [26,27]. CD71, also known as the transferrin receptor, may influence the function and differentiation of immune cells in iron deficiency [28,29]. In our study, experimental DEA was established and appropriate responses were obtained in all four immunohistochemical methods examined (SLC11a, IRE1, WNT, and CD71). Although positive responses were obtained in the treated groups, the most optimal results were obtained in Group 7. Statistically significant differences were found in the groups receiving probiotics compared to the group receiving DEA medical treatment (Fe^2+^) (Table 6). There is not enough literature information on the effects of probiotics on these molecules and further studies are needed.

Functional foods are defined as products that provide health benefits beyond their traditional nutritional value [30]. There are many studies that have been conducted to increase iron absorption from foods through probiotics. In a study with the addition of probiotics to fruit drinks in young women, it was found that iron absorption from fruit drinks could be increased by approximately 50% [6]. Similarly, in a study conducted by giving a capsule containing *L. plantarum* 299v with breakfast, an increase of about 50% in iron absorption was found [7]. A study was conducted on wheat porridge and breakfast wheat buns with *L. plantarum* 299v and an increase in iron absorption was found [28,31]. Hb levels were higher in children with RIA who received iron preparations with lactobacilli than those who received iron preparations alone [29]. In the literature review, no study on probiotic supplementation with molasses containing high amounts of iron was observed. Our study results confirm the positive effects of probiotics on iron absorption and metabolism in accordance with the literature. In a randomized, double-blind, controlled trial for the treatment of iron deficiency in children, the use of low-dose ferrous sulfate was compared with or without probiotics (LP299v). There was no significant difference in the increase in serum ferritin in children receiving LP299v compared with controls [7]. In healthy female volunteers of childbearing age, iron absorption was examined in a breakfast meal given with capsules with and without Lp299v. Iron absorption was significantly lower in the group without Lp299v than in the group with Lp299v [29]. In our study, significant improvement was found in hepcidin and ferritin values.

The mechanism of action of probiotics on iron absorption has not been fully elucidated. The European Food Safety Authority has reported that probiotics improve iron absorption [32]. It has been shown that molecules released by probiotic bacteria increase absorption by converting Fe ^3+^ to Fe ^2+^ in the presence of duodenal cytochrome b Reductase 1 [33]. In addition, probiotics may interact with intestinal walls and increase absorption as a result of mucosal healing [34]. According to another theory, *L. fermentum* and *B. breve* have been observed to have dense small iron oxide nanoparticles on their outer surfaces. The addition of these bacteria to iron therapy may increase the bioavailability of nanoparticles and provide relief from stomach diseases [34,35]. In controlled experiments, Streptococcus thermophilus, Lactobacillus fermentum, and Lactobacillus plantarum were reported to affect the expression and activity of key proteins. While Lactobacillus fermentum restored decreased DMT1 and duodenal cytochrome b levels in anemic rat models and human cell lines, *p*-hydroxyphenyllactic acid produced by the strain was observed to increase DMT1 activity through ferric iron reduction (34). Furthermore, *Lactobacillus plantarum* 299v was observed to increase duodenal cytochrome b expression by 24% (*p* = 0.027) in human intestinal Caco-2 cultures [31]. Lactic acid-forming bacteria, including lactobacilli, are thought to increase dietary iron bioavailability through several mechanisms such as reducing intestinal pH, shifts in gut microbiota metabolism and metabolite formation, and promotion of anti-inflammatory immunomodulation [9]. Generally, mucins bind to iron owing to the acidic conditions in the stomach, which helps in maintaining it in a soluble state for later uptake in the alkaline conditions of the duodenum. *p*-hydroxyphenyllactic acid, a metabolite produced by Lactobacillus fermentum, increases iron absorption through the DMT 1 transporters from enterocytes by reducing the Fe^3+^ to Fe^2+^. A similar situation is valid for molecules such as ascorbic acid, acetic acid, citric acid, propionic acid, etc. [36]. In our study, intestinal pH could not be analyzed, but we think that the probiotics we used increased iron absorption by the mechanisms in the examples given above.

There are several substantial limitations of this study. The main limitation is that the rate of iron absorption by probiotic supplementation could not be determined. In addition, liver histopathology, which is necessary for the diagnosis of NAFLD, could not be performed. In future studies, it would be useful to indicate liver histopathology and the rate of increase in absorption. In our study, as in the referenced studies, the rats were given 1 × 10^8^ probiotic suj. Although different species were used, studies with different doses of probiotic preparations are needed. Also, our study is a pilot study that reveals the effects of molasses and probiotics on iron absorption in anemia caused by experimentally induced iron deficiency. The results of this study will shed light on more comprehensive studies to be carried out with molecular and clinical analysis methods in the future. However, further research is required in the future to transfer our results directly to humans.

## 7. Conclusions

Although iron absorption in molasses was increased with probiotic supplementation, no significant difference was detected in serum iron panel values when the probiotic groups were compared within themselves. In addition, probiotics can be considered as a clinical tool to optimize dietary iron bioavailability without creating an additional gastrointestinal burden to improve iron status.

## Figures and Tables

**Table 1:** Regimens given to rats with iron deficiency anemia.

	Group 1 (Classic) (*n* = 6)	Group 2 (Iron Purified) (*n* = 6)	Group 3 (Molasses) (*n* = 7)	Group 4 (Fe^3+^) (*n* = 6)	Group 5 (LP299v.) (*n* = 7)	Group 6 (Polyprobiotic) (*n* = 7)	Group 7 (Polyprobiotic + vit) (*n* = 7)
Classic feed	+	−	−	−	−	−	−
Iron purified feed	−	+	+	+	+	+	+
Molasses	−	−	−	+	+	+	+
Fe^3+^	−	−	+	−	−	−	−
*Lactobacillus plantarum* 299v.	−	−	−	−	+	−	−
Polyprobiotic	−	−	−	−	−	+	−
Polyprobiotic + vitamin complex	−	−	−	−	−	−	+

**Table 2:** (**a**) Grading score of the mucosal damage score (MHDS); (**b**) Immun-Positivity Score.

	a	b
Grade	Sequence of Morphological Changes	Results
**0**	Normal mucosal villi	Less Than 5%
**1**	Development of subepithelial Gruenhagen’s space, usually at the apex of the villus; often with capillary congestion.	Between 5–25%
**2**	Extension of the subepithelial space with moderate liftingof epithelial layer from the lamina propria.	Between 25–50%
**3**	Mild crypt loss (four crypts in each villus) accompanying villar fusion	Between 51–75%
**4**	Moderate crypt loss (fewer than four crypts in each villus) accompanying villar fusion	More than 75%
**5**	Severe crypt loss (fewer than three crypts in each villus)	

**Table 3:** Data on metabolic status of experimental animals.

Group	Mean	SS	Min	Max	*p*	Group	Mean	SS	Min	Max	*p*
Body Weight (g)						Glucose (mg/dL)					
**Classic**	321.7	52.9	260	382	0.83	**Classic**	157.7	22	129	191	0.23
**Iron purified**	309.8	70.2	244	382		**Iron purified**	164.2	25.7	126	199	
**Molasses**	305.1	69.4	226	386		**Molasses**	203	51.7	143	295	
**Fe^3+^**	316	80.7	230	403		**Fe^3+^**	170.8	29.6	133	201	
**LP299v.**	286.6	69.5	228	399		**LP299v.**	150.3	40.8	97	221	
**Polyprobiotic**	304.4	78.6	246	424		**Polyprobiotic**	144.1	28.1	97	181	
**Polyprobiotic + vit**	325.7	43.2	281	380		**Polyprobiotic + vit**	162	38.9	102	202	
**Triglyceride (mg/dL)**						**Cholesterol (mg/dL)**					
**Classic**	78.5	11.5	64	93	0.02	**Classic**	69.8	18.4	45	101	0.14
**Iron purified**	33	1.8	27	39		**Iron purified**	71.5	4.4	57	88	
**Molasses**	44.3	3.7	32	59		**Molasses**	92.3	10.7	65	142	
**Fe^3+^**	45.8	5.7	29	66		**Fe^3+^**	74.2	7.8	55	99	
**LP299v.**	47.4	5.1	34	76		**LP299v.**	85.6	8.8	57	115	
**Polyprobiotic**	42.9	5.5	25	65		**Polyprobiotic**	74.4	2.8	65	87	
**Polyprobiotic + vit**	38.3	2.9	29	51		**Polyprobiotic + vit**	63.1	4.1	54	84	
**LDL Cholesterol (mg/dL)**						**HDL Cholesterol (mg/dL)**					
**Classic**	16.7	5.2	11	24	0.36	**Classic**	43	11.5	33.6	64	0.08
**Iron purified**	18.5	3.4	15	24		**Iron purified**	46.5	7.8	35.9	58.1	
**Molasses**	25.6	10.6	15	43		**Molasses**	57.8	17.9	39.9	89.4	
**Fe^3+^**	17.2	6.3	11	25		**Fe^3+^**	47.8	11.5	37	63.3	
**LP299v.**	22.7	10.3	9	36		**LP299v.**	53.5	12.1	38.3	67.5	
**Polyprobiotic**	18.3	6.1	8	27		**Polyprobiotic**	47.6	4.4	43	56.5	
**Polyprobiotic + vit**	15.7	3.7	11	22		**Polyprobiotic + vit**	39.9	7.3	33.7	54.7	
**ALT (IU/L)**						**AST (IU/L)**					
**Classic**	39.5	9.7	31	55	0.06	**Classic**	146.8	39	102	215	0.94
**Iron purified**	25.2	7.2	19	37		**Iron purified**	159.3	54.4	110	258	
**Molasses**	29.1	8.6	22	44		**Molasses**	150.4	34	102	187	
**Fe^3+^**	28.2	2.4	26	31		**Fe^3+^**	148.7	29.2	116	192	
**LP299v.**	28	8.4	15	39		**LP299v.**	177	115.2	101	425	
**Polyprobiotic**	30.4	5	21	37		**Polyprobiotic**	155	18.2	130	188	
**Polyprobiotic + vit**	36.6	10.1	29	57		**Polyprobiotic + vit**	178.3	69.3	110	314	

**Table 4:** Laboratory data on iron metabolism of experimental animals.

Group	Mean	SS	Min	Max	*p*	Group	Mean	SS	Min	Max	*p*
Iron (mg/dL)						UIBC (mg/dL)					
**Classic**	245.7	98.3	120	346	0.41	**Classic**	316.83	52.121	214	348	0.12
**Iron purified**	182.0	66.8	112	265		**Iron purified**	325.67	25.264	275	341	
**Molasses**	276.7	84.0	168	355		**Molasses**	305.14	62.715	172	346	
**Fe^3+^**	211.7	89.6	117	317		**Fe^3+^**	312.83	45.146	228	344	
**LP299v.**	234.7	54.7	136	291		**LP299v.**	310.43	37.411	252	343	
**Polyprobiotic**	224.7	68.3	151	322		**Polyprobiotic**	323.43	30.794	260	345	
**Polyprobiotic + vit**	221.1	67.3	138	319		**Polyprobiotic + vit**	343.14	1676	342	346	
**RBC (10^3^/µL)**						**Hb (g/dL)**					
**Classic**	7.7	0.7	6.7	8.7	0.34	**Classic**	14.8	1.2	13.4	16.6	0.4
**Iron purified**	7.0	0.4	6.6	7.6		**Iron purified**	13.0	0.7	12.2	14.2	
**Molasses**	7.2	0.4	6.4	7.6		**Molasses**	13.5	0.8	12.5	14.6	
**Fe^3+^**	7.2	0.7	6.6	7.8		**Fe^3+^**	13.2	0.4	12.7	13.7	
**LP299v.**	7.4	0.7	6.5	8.0		**LP299v.**	14.2	1.2	11.8	15.2	
**Polyprobiotic**	7.3	0.8	5.8	8.3		**Polyprobiotic**	13.6	1.7	10.1	15.1	
**Polyprobiotic + vit**	7.3	0.5	6.4	7.9		**Polyprobiotic + vit**	13.8	0.6	13.1	14.6	
**Ferritin (ng/mL)**						**Hepcidin (ng/mL)**					
**Classic**	21.9	1.28	20.3	23.4	0.03	**Classic**	302.7	34.4	275.8	366.5	0.02
**Iron purified**	17.7	1.1	15.8	18.7		**Iron purified**	285.0	35.9	253.0	338.5	
**Molasses**	20.2	2.2	16.9	23.8		**Molasses**	301.7	43.1	244.8	371.2	
**Fe^3+^**	16.5	2.6	14.1	20.6		**Fe^3+^**	239.5	35.3	210.4	295.5	
**LP299v.**	19.3	1.7	16.7	21.4		**LP299v.**	336.2	116.6	250.9	592.9	
**Polyprobiotic**	19.8	1.8	16.9	22.1		**Polyprobiotic**	341.7	34.6	310.9	404.7	
**Polyprobiotic + vit**	19.2	2.1	15.6	21.5		**Polyprobiotic + vit**	301.9	37.9	250.2	358.7	

**Table 5:** Mucosal histopathological damage score (MHDS, median—25–75% interquartile range).

Group	MHDS
**Classic**	0 (0–0)
**Iron purified**	3.5 (3–4) **^a^**
**Molasses**	2 (2–2) **^a,b^**
**Fe^3+^**	2 (1–2) **^a,b^**
**LP299v.**	2 (1–2) **^a,b^**
**Polyprobiotic**	1 (1–2) **^a,b^**
**Polyprobiotic + vit**	1 (1–1) **^a,b,c,d^**

^a ^*p* = 0.001 versus Group 1; ^b ^*p* = 0.001 versus Group 2; ^c ^*p* = 0.001 versus Group 3; ^d ^*p* = 0.001 versus Group 4; Kruskal–Wallis test and Mann–Whitney U test with Bonferroni corrections

**Table 6:** Immunopositive numerical density measurement (mm^3^) data.

Group	Slc11a	IRE1	WNT2	CD71
**Classic**	62.6 ± 4.02	11.7 ± 2.68	6.9 ± 2.75	4.7 ± 0.65
**Iron purified**	16.8 ± 4.19 **^a^**	66.5 ± 92.26 **^a^**	28.4 ± 6.26 **^a^**	22.95 ± 8.89 **^a^**
**Molasses**	38.3 ± 3.46 **^a,b^**	20.4 ± 3.81 **^b^**	12.7 ± 2.34 **^a,b^**	14.6 ± 1.78 **^a,b^**
**Fe^3+^**	41.65 ± 3.99 **^a,b^**	21.30 ± 4.81 **^b,g^**	12.95 ± 1.57 **^a,b,g^**	15.45 ± 1.79 **^i,b,d^**
**LP299v.**	44.15 ± 5.73 **^a,b,c^**	17.25 ± 1.83 **^b^**	13.45 ± 1.67 **^a,b^**	9.5 ± 2.21 **^a,b,d,e^**
**Polyprobiotic**	52.2 ± 4.2 **^a,b,e,f^**	10.4 ± 2.98 **^b^**	10.55 ± 1.87 **^h,b^**	10.35 ± 2.27 **^h,b,c,e^**
**Polyprobiotic + vit**	55.85 ± 3.44 **^a,b,e,f^**	9.15 ± 2.13 **^b^**	9.3 ± 2.15 **^b,j,k,f^**	9.2 ± 2.91 **^l,b,d,e^**

**^a ^***p* = 0.001 versus Group 1; **^b^** *p* = 0.001 versus Group 2; **^c^** *p* = 0.01 versus Group 3; **^d^** *p* = 0.001 versus Group 4; **^e^** *p* = 0.001 versus Group 4; **^f^** *p* = 0.001 versus Group 5; **^g^** *p* = 0.002 versus Group 2; **^h^** *p* = 0.005 versus Group 1; **^i^** *p* = 0.013 versus Group 3; **^j^** *p* = 0.005 versus Group 4; **^k^** *p* = 0.003 versus Group 1; **^l^** *p* = 0.007 versus Group 1; one-way ANOVA test with Bonferroni test.

## Data Availability

The data presented in this paper are available upon request from the corresponding author. The data are not publicly available due to privacy and ethical restrictions.
